# An evaluation of methods correcting for cell-type heterogeneity in DNA methylation studies

**DOI:** 10.1186/s13059-016-0935-y

**Published:** 2016-05-03

**Authors:** Kevin McGregor, Sasha Bernatsky, Ines Colmegna, Marie Hudson, Tomi Pastinen, Aurélie Labbe, Celia M.T. Greenwood

**Affiliations:** McGill University, Department of Epidemiology, Biostatistics, and Occupational Health, 1020 Pine Ave. West, Montréal, H3A 1A2 QC Canada; Lady Davis Research Institute, Jewish General Hospital, 3755 Chemin de la Côte Sainte Catherine, Montréal, H3T 1E2 QC Canada; Division of Rheumatology, Jewish General Hospital, Montréal, QC Canada; McGill University and Genome Quebec Innovation Centre, McGill University, Montréal, QC Canada; Department of Human Genetics, McGill University, Montréal, QC Canada; Department of Medicine, McGill University, Montréal, QC Canada; The Research Institute of the McGill University Health Centre, Montréal, QC Canada; Department of Psychiatry, McGill University, Montréal, QC Canada; The Douglas Mental Health University Institute, Verdun, QC Canada

**Keywords:** DNA methylation, Cell-type mixture, Deconvolution, Matrix decomposition

## Abstract

**Background:**

Many different methods exist to adjust for variability in cell-type mixture proportions when analyzing DNA methylation studies. Here we present the result of an extensive simulation study, built on cell-separated DNA methylation profiles from Illumina Infinium 450K methylation data, to compare the performance of eight methods including the most commonly used approaches.

**Results:**

We designed a rich multi-layered simulation containing a set of probes with true associations with either binary or continuous phenotypes, confounding by cell type, variability in means and standard deviations for population parameters, additional variability at the level of an individual cell-type-specific sample, and variability in the mixture proportions across samples. Performance varied quite substantially across methods and simulations. In particular, the number of false positives was sometimes unrealistically high, indicating limited ability to discriminate the true signals from those appearing significant through confounding. Methods that filtered probes had consequently poor power. QQ plots of *p* values across all tested probes showed that adjustments did not always improve the distribution. The same methods were used to examine associations between smoking and methylation data from a case–control study of colorectal cancer, and we also explored the effect of cell-type adjustments on associations between rheumatoid arthritis cases and controls.

**Conclusions:**

We recommend surrogate variable analysis for cell-type mixture adjustment since performance was stable under all our simulated scenarios.

**Electronic supplementary material:**

The online version of this article (doi:10.1186/s13059-016-0935-y) contains supplementary material, which is available to authorized users.

## Background

DNA methylation is an important epigenetic factor that modulates gene expression through the inhibition of transcriptional proteins binding to DNA [1]. Examining the associations between methylation and phenotypes, either at a few loci or epigenome-wide (i.e. the Epigenome-Wide Association Study (EWAS) [2]) is an increasingly popular study design, since such studies can improve understanding of how the genome influences phenotypes and diseases. However, unlike genetic association studies, where the randomness of Mendelian transmission patterns from parents to children enables some inference of causality for associated variants, results from EWAS studies can be more difficult to interpret.

The choices of tissue for analysis and time of sampling are crucial, since methylation levels vary substantially across tissues and time. Methylation plays a large role in cellular differentiation, especially in regulatory regions [3, 4], and methylation patterns are largely responsible for determining cell-type-specific functioning, despite the fact that all cells contain the same genetic code [5].

Ideally, methylation would be measured in tissues and cells of most relevance to the phenotype of interest, but in practice such tissues may be impossible to obtain in human studies. Many accessible tissues for DNA methylation studies, such as saliva, whole blood, placenta, adipose, tumors, or many others, will contain mixtures of different cell types, albeit to varying degrees. Hence, the measured methylation levels represent weighted averages of cell-type-specific methylation levels, with weights corresponding to the proportion of the different cell types in a sample. However, cell-type proportions can vary across individuals, and can be associated with diseases or phenotypes [6]. For example, individuals with autoimmune disease are likely to have very different proportions of autoimmune cells in their blood than non-diseased individuals [7–11], synovial and cartilage cell proportions differ between rheumatoid arthritis patients and controls [12], and associations with age have been consistently reported [13]. Hence, variable cell-type-mixture proportions can confound relationships between locus-specific methylation levels and phenotypes, since these proportions are associated both with phenotype and with methylation levels [14].

In situations potentially subject to confounding, although less biased estimates of association can be obtained by incorporating the confounding variable as a covariate, this is not a perfect solution, since it may not be possible to distinguish lineage differences [14] or to estimate accurately the proportions of each cell type in a tissue sample [15, 16]. Initial studies of associations between DNA methylation and phenotypes largely ignored this potential confounding factor, which may have led to biased estimates of association and failure to replicate findings [17, 18].

However, in parallel with the increasing prevalence of high-dimensional methylation studies, a number of methods that can account for this potential confounding of methylation–phenotype associations have been developed or adapted from other contexts. Among those developed specifically for methylation data (Ref-based [19], Ref-free [20], CellCDec [21], and EWASher [22]), the first two were proposed by the same author (Houseman), but the first of these requires an external reference data set. Other methods were proposed in more general contexts where confounding does not necessarily result from cell-type mixtures yet is still of concern; many of these rely on some implementation of matrix decompositions (SVA [23], ISVA [24], Deconfounding (Deconf) [25], and RUV [26, 27]).

Although there are numerous similarities between the approaches, there remain some fundamental differences in terms of limitations and performance. An unbiased comparison of methods has been difficult since true cell-type mixture proportions are unknown, replications using alternative technologies such as targeted pyrosequencing do not lead to genome-wide data where cell-type proportions can be estimated, and new methods have tended to be compared with only a few other approaches. Since the problem of confounding plagues all researchers in this field, a careful comparison of existing methods correcting for cell-type heterogeneity is essential, and this is the objective of our paper.

In an ongoing study of incident treatment-naive patients with one of four systemic autoimmune rheumatic diseases (SARDs), whole-blood samples were taken at presentation, and immune cell populations (purity >95 %) were sorted from the peripheral mononuclear cells (PBMCs) of these patients. Analysis of DNA methylation was then performed with the Illumina Infinium HumanMethylation450 BeadChip (450K) on the cell-separated data. These data provide a unique and valuable opportunity to compare the performance of methods for cell-type mixture adjustments. We present here the results of an extensive simulation study where we remixed the cell-separated methylation profiles to incorporate variable mixture proportions and confounding of associations, and we then compare performance of eight different methods of adjustment. We also compare the ease of use of each method, and provide an R script allowing for easy implementation of several of the best-performing methods. As far as we are aware, this is the first study to compare such an extensive set of methods in a simulation based on cell-separated data.

## Results

### Patients and original methylation profiles

Whole-blood samples were obtained from patients with incident treatment-naive rheumatoid arthritis (*n*=11), systemic lupus erythematosus (*n*=9), systemic sclerosis (*n*=14), and idiopathic inflammatory myositis (*n*=3). Several control samples were available as well (*n*=9). CD4, CD19, and CD14 subpopulations were sorted from PBMCs by magnetic cell isolation and cell separation (MACS) sorting (see “Methods”). The purity of the isolated populations was confirmed by flow cytometry. Only in samples with a purity >95 % were methylation profiles assessed using the Illumina Infinium HumanMethylation 450 BeadChip on the separate cell populations. Our simulation and results are based primarily on 46 patients for whom cell-sorted methylation profiles were available for both CD4 ^+^ T lymphocytes and CD14 ^+^ monocytes.

**Fig. 1:**
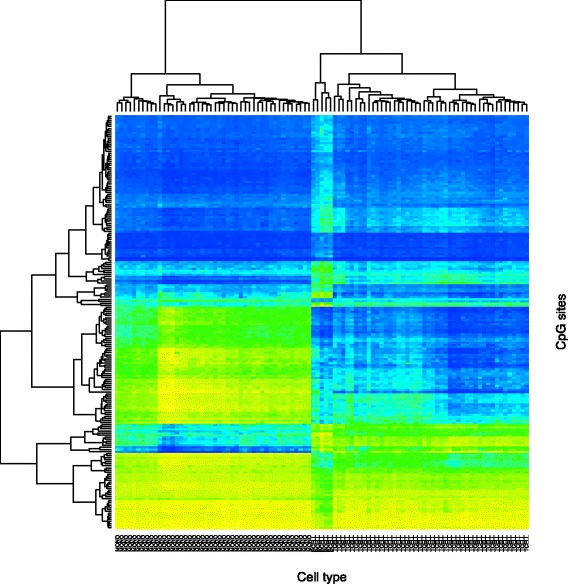
Clustered heat map showing patterns of methylation in 46 SARD samples (columns) and 200 CpG sites (rows). The sites were selected to highlight the methylation differences between cell types. Consequently, the samples cluster by cell type: monocytes, B cells, and then T cells

The heat map in Fig. 1 shows some representative patterns of methylation in the SARD samples across CD14 ^+^ monocytes, CD4 ^+^ T cells, and CD19 ^+^ B cells (this latter cell type was not available for all patients), at 200 CpG sites that were selected because of the inter-cell-type differences. The figure demonstrates that there are sizeable differences in methylation levels between cell types, and it follows that small variations in the proportions of these component cell types in a mixed tissue sample can lead to great difficulties in interpreting any phenotype-associated results.

### Multi-layer simulation design

We implemented a rich simulation design, based on the SARD methylation data. This simulation contains random sources of variability at multiple levels, including both variability of population mean parameters as well as variability at individual-level parameters. Starting with the observed cell-separated methylation profiles for T cells and monocytes from the SARD data, we simulated a number of probes to have associations directly with the phenotype, and we induced confounding by combining the two cell types in proportions that vary across individuals. Although this simulation design is complex and depends on a large number of parameters, it allows substantial flexibility in specifying consistency or variability between cell types, individuals, or probes, and easily allows us to create realistic and pathological situations in the same framework.

Let *i*=1,…,*n* where *n*=46 denote the individuals in the SARD data. In brief, the simulation proceeds as follows (see “Methods” for more details):

1. Select a set of *S* CpG sites where “true” associations with a phenotype will be generated by our simulation; we refer to these CpGs as differentially methylated sites (DMSs).
2. Generate a phenotype, either binary (disease or no disease) or continuous.
3. For any probe not in the DMS set, the cell-type-specific methylation values are the observed values from the real data.
4. For a probe in the DMS set, a randomly generated quantity is added to the observed cell-type-specific level of methylation, in a way that depends on the phenotype.
5. The cell-type-specific methylation values are mixed together in proportions that vary depending on the phenotypes.

Over all DMSs, one would expect to see a range of positive and negative associations with the phenotype. In step 4, we allow these associations to differ between cell types in order to specify an association between change in methylation and cell type. After having specified each of the site- and cell-type-specific associations, we then add between-subject variability to each site. The final step, step 5, leads to methylation proportions as they would appear had the mixed tissue been analyzed directly.

After simulating the data, we then test for association between the phenotype and the methylation levels in the mixed data at each probe. We compare the *p* values obtained from these tests of association across eight simulation scenarios and eight different methods for cell-type adjustment. Some of the DMSs were simulated to have very small effects and therefore, statistical tests of association may not be significant. On the other hand, since the cell-type proportions vary with phenotype, this can lead to non-DMSs showing spurious associations with the phenotype. The notation for the key parameters is given in Table 1, and the parameter choices across different simulation scenarios are summarized in Table 2.

**Table 1:** Fixed parameters in the simulation design

Parameter	Description
*S*	Number of CpGs chosen to be associated with phenotype in simulation
*μ*_*k*_	Mean of the cell-type-specific DMS effects for cell type *k*, over all *S* DMSs
*σ*_*k*_	Standard deviation of cell-type-specific DMS effects for cell type *k*, over all *S* DMSs
*σ*_*jk*_	Variability of individual deviations at probe j in cell type k
$\alpha ^{(0)} = \left (\alpha _{1}^{(0)}, \alpha _{2}^{(0)}\right)^{\top }$	Expected proportion of the mixture from cell types 1 and 2 when the phenotype *z*_*i*_ is zero.
$\alpha ^{(Z)} = \left (\alpha _{1}^{(Z)}, \alpha _{2}^{(Z)}\right)^{\top }$	Average cell-type mixture proportions for cell types 1 and 2 for subjects with phenotype level *Z* (continuous or binary)
*ρ*	Precision of simulated cell mixture distributions. A greater value corresponds to more clearly defined differences in cell-type proportions with respect to the phenotype

**Table 2:** Parameter choices for the simulation scenarios

	Simulation scenario	(*μ*_1_,*μ*_2_)	(*σ*_1_,*σ*_2_)	*σ*_*jk*_	*ρ*	*α* ^(0)^	*α* ^(1)^−*α* ^(0)^ ^a^
1	Distinct differences	(−0.05,0.5)	(0.05, 0.75)	Unif(0.1, 2)	100	$\left [\begin {array}{c} 0.57 \\[-5pt] 0.43 \end {array}\right ]$	$\left [\begin {array}{r}0.08 \\[-5pt] -0.08\end {array}\right ] $
2	No confounding	(0.25, 0.25)	(0.5, 0.5)	0.1	100	$\left [\begin {array}{c} 0.57 \\[-5pt] 0.43 \end {array}\right ]$	$\left [\begin {array}{r}0.08 \\[-5pt] -0.08 \end {array}\right ]$
3	Opposite effects	(−0.75,0.75)	(0.1, 0.1)	0.1	100	$\left [\begin {array}{c}0.57 \\[-5pt] 0.43 \end {array}\right ]$	$\left [\begin {array}{r}0.08 \\[-5pt] -0.08 \end {array}\right ]$
4	High precision	(0.3, 0.1)	(0.1, 0.1)	0.1	200	$\left [\begin {array}{c}0.57 \\[-5pt] 0.43 \end {array}\right ]$	$\left [\begin {array}{r}0.08 \\[-5pt] -0.08 \end {array}\right ]$
5	Low precision	(0.3, 0.1)	(0.1, 0.1)	0.1	10	$\left [\begin {array}{c}0.57 \\[-5pt] 0.43 \end {array}\right ]$	$\left [\begin {array}{r}0.08 \\[-5pt] -0.08 \end {array}\right ]$
6	Continuous phenotype	(−0.05,0.25)	(0.05, 0.15)	0.1	100	$\left [\begin {array}{c}0.57 \\[-5pt] 0.43 \end {array}\right ]$	$\left [\begin {array}{r}0.03 \\[-5pt] -0.03 \end {array}\right ]$
7	Few assoc. sites	(1, 0.95)	(0.05, 0.05)	Unif(0.1, 2)	100	$\left [\begin {array}{c}0.57 \\[-5pt] 0.43 \end {array}\right ]$	$\left [\begin {array}{r}0.08 \\[-5pt] -0.08 \end {array}\right ]$
8	Many assoc. sites block 1 ^b^	(0.1,0.4)	(0.01, 0.01)	Unif(0.1, 2)	100	$\left [\begin {array}{c}0.57 \\[-5pt] 0.43 \end {array}\right ]$	$\left [\begin {array}{r}0.08 \\[-5pt] -0.08 \end {array}\right ]$
	Many assoc. sites block 2 ^c^	(0.2,0.7)	(0.01, 0.01)	Unif(0.1, 2)	100	$\left [\begin {array}{c}0.57 \\[-5pt] 0.43 \end {array}\right ]$	$\left [\begin {array}{r}0.08 \\[-5pt] -0.08 \end {array}\right ]$

*k*=1 corresponds to monocytes, and *k*=2 corresponds to CD4 ^+^ T cells

^a^Average change in cell-type proportion for unit increase in phenotype

^b^Background correlation for block 1 was 0.4

^c^Background correlation for block 2 was 0.5

### Scenario 1: DMSs have differences in both means and variances between cell types

In our first simulation scenario (Table 2), we chose to specify distinct differences in the strength and distribution of the methylation–phenotype associations (DMS) for the two cell types, with a binary phenotype. Differences in the DMS distributions include both direction of the effects as well as the amount of variability across sites and individuals; Additional file 1: Figure S1 displays histograms of the 500 simulated values of the DMS means *μ*_*jk*_ for the two cell types, showing the substantial differences between these two distributions.

**Fig. 2:**
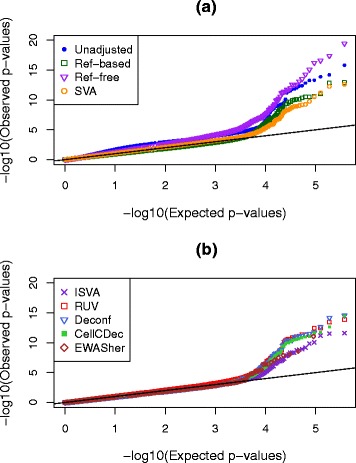
QQ plots showing distributions of *p* values in simulation scenario 1 where the true effects in the different cell types have very distinct distributions. Results are shown with no adjustment for cell-type mixture as well as with eight other methods; these are split across two panels, (**a**) and (**b**), to clarify the display

In this scenario, we would expect that an analysis not taking cell type into consideration should result in many *p* values that are smaller than expected, or equivalently a greatly inflated slope in a *p* value QQ plot, due to the strong confounding built into the simulation design. After analyzing for all probes with unadjusted data, we repeated the epigenome-wide analysis with eight popular or newly developed adjustment methods (see “Methods”). QQ plots for these eight methods as well as the uncorrected analysis can be seen in Fig. 2. Examination of the left-hand side of this plot (*x*-axis smaller than about 3.5) shows that there is, indeed, a genome-wide inflation of *p* values in the analysis uncorrected for cell-type mixture. Encouragingly, most methods do a fairly good job in correcting for the confounding, since the corrected QQ plots are close to the line of expectation up until the tails of each set of *p* values. The reference-free method, however, continues to display inflation even after correction.

Several numeric performance metrics can be seen in Table 3 for this simulation scenario. One of these metrics is the genomic inflation factor (GIF) [28], which is the slope of the lines seen in Fig. 2 after removing the 500 DMSs. The unadjusted GIF was 1.6, indicating a substantial inflation of significance across all *p* values, but after adjustment most values are quite close to 1.0, as would be expected in the absence of any confounding. The Ref-based, EWASher, and Deconf methods have slopes slightly less than 1.0, implying possible over-correction.

**Table 3:** Performance metrics under simulation scenario 1 (distinct associations between cell types)

Method	Number of false positives	Power	KS	GIF	$\hat {K}$ ^a^
Unadjusted	248	0.208	0.168	1.60	–
Ref-based	14	0.146	0.026	0.92	–
Ref-free	375	0.248	0.097	1.33	13
SVA	32	0.124	0.021	1.05	10
ISVA	14	0.134	0.004	0.97	12
EWASher	3	0.036	0.044	0.92	–
CellCDec	7	0.094	0.008	0.98	–
Deconf	13	0.178	0.023	0.93	–
RUV	36	0.170	0.018	1.04	43

*KS* Kolmogorov–Smirnov statistic, *GIF* genomic inflation factor

^a^Estimated latent dimension

Since we know which 500 sites were generated to be truly DMSs, Table 3 reports both the power and the number of false positives (NFP). We declare a CpG site to be significant if the *p* value falls below the fixed value 10^−4^. This table also shows a measure of performance based on the Kolmogorov–Smirnov (KS) test for whether the *p* value distribution matches the expected uniform distribution. Of course, the KS test assumes independence of all the individual tests, and therefore, we are not using this test for inference, but simply as a measure of deviation where smaller values imply less deviation.

For this simulation (scenario 1 with distinct association distributions in the two cell types), all methods except the reference-free method achieved a greater reduction in the NFP than the unadjusted analysis for the non-associated probes. Although the power (sensitivity) for all the methods appears very low, many of the simulated effect sizes at the chosen DMSs were very small, and nevertheless the rankings of the different methods are still informative. Additional file 1: Figure S1 shows the simulated means of the cell-type distributions for the 500 probes; subsequently, additional random errors were introduced at the level of each individual leading to substantial variability in the realized methylation differences. The power of most methods was slightly less than that of the unadjusted analysis, except for Ref-free and EWASher. The power for EWASher is extremely poor; this method removes probes with very high or very low levels of methylation prior to constructing the components, and hence, many DMS probes are not even included in its analyses. Results for the Ref-free power must be interpreted cautiously since the type 1 error is so substantially elevated for this method. The KS statistic confirms the conclusions obtained from other metrics, showing small values for most methods except for the unadjusted data and the Ref-free method.

### Scenario 2: no confounding

It is also of interest to examine performance when there is no confounding. By simulating data with the same cell-type-specific means and variances in both cell types (scenario 2 in Table 2), the unadjusted analysis should not be subject to any bias. As expected, Table 4 shows low NFP and good power for the unadjusted method, and similar results are obtained for CellCDec, Deconf, and Ref-based. In Additional file 1: Figure S2, it can be seen that the unadjusted results lie very close to the line of expectation, apart from the tail of the distribution where the DMSs predominate. It is interesting to note that SVA, RUV, Ref-free, and in particular ISVA display high NFP, implying that far too many DMS probes are being inferred. Despite that no confounding was simulated, the GIF for the unadjusted data is slightly inflated; in fact, after adjustment, the GIF increases for Ref-free and ISVA. In contrast, the GIF is less than 1 for CellCDec, Deconf, and the Ref-based methods, implying some over-correction.

**Table 4:** Performance metrics under simulation scenario 2 (no confounding)

Method	Number of false positives	Power	KS	GIF	$\hat {K}$
Unadjusted	38	0.642	0.059	1.09	–
Ref-based	13	0.594	0.030	0.90	–
Ref-free	366	0.704	0.058	1.25	14
SVA	79	0.646	0.015	1.06	10
ISVA	408	0.654	0.061	1.27	15
EWASher	0	0.126	0.066	0.83	–
CellCDec	14	0.650	0.021	0.93	–
Deconf	50	0.652	0.031	0.92	–
RUV	134	0.640	0.007	1.00	38

*KS* Kolmogorov–Smirnov statistic, *GIF* genomic inflation factor

### Scenario 3: opposite effects in different cell types

To investigate a case of severe differential effects, in scenario 3, the cell-type-specific means *μ*_*k*_ were selected to have opposite signs in the two cell types. In this case, the mixed sample can have small DMS effects, since the two cell-type-specific effects may cancel each other. Confirming this expectation, there is no inflation of the test statistics in the unadjusted data (GIF = 0.97, Table 5). Like the previous scenarios, we see very poor power and over-correction with EWASher, and extremely inflated NFP with the Ref-free method (Additional file 1: Figure S3). Small power improvements over the unadjusted analysis can be seen when using any of the other methods.

**Table 5:** Performance metrics under simulation scenario 3 (opposite effects)

Method	Number of false positives	Power	KS	GIF	$\hat {K}$
Unadjusted	5	0.490	0.041	0.97	–
Ref-based	1	0.506	0.036	0.87	–
Ref-free	176	0.608	0.053	1.19	14
SVA	37	0.562	0.003	1.00	10
ISVA	49	0.558	0.006	1.01	14
EWASher	0	0.128	0.092	0.74	–
CellCDec	3	0.502	0.033	0.88	–
Deconf	2	0.522	0.035	0.89	–
RUV	23	0.532	0.012	1.00	37

*KS* Kolmogorov–Smirnov statistic, *GIF* genomic inflation factor

### Scenarios 4 and 5: altered precision simulations

Two scenarios were generated where we changed the precision of the individuals’ cell-type distributions between cases and controls. That is, a higher precision corresponds to a more pronounced separation in the cell-type distributions between cases and controls, while a lower precision makes the two distributions more difficult to distinguish. Here, both T cells and monocytes were chosen to have distinct, positive net association with the phenotype; however, the precision parameter, *ρ*, from the Dirichlet distribution was varied such that *ρ*=200 for high precision and *ρ*=10 for low precision. QQ plots are shown in Additional file 1: Figure S4 (high precision) and S5 (low precision), and numeric metrics are in Tables 6 and 7.

**Table 6:** Performance metrics under simulation scenario 4 (high precision)

Method	Number of false positives	Power	KS	GIF	$\hat {K}$
Unadjusted	368	0.600	0.142	1.32	–
Ref-based	64	0.460	0.043	1.04	–
Ref-free	437	0.694	0.101	1.36	13
SVA	79	0.534	0.032	1.09	11
ISVA	198	0.522	0.062	1.20	14
EWASher	16	0.072	0.038	0.94	–
CellCDec	54	0.546	0.031	1.05	–
Deconf	58	0.520	0.014	1.02	–
RUV	155	0.568	0.051	1.15	32

*KS* Kolmogorov–Smirnov statistic, *GIF* genomic inflation factor

**Table 7:** Performance metrics under simulation scenario 5 (low precision)

Method	Number of false positives	Power	KS	GIF	$\hat {K}$
Unadjusted	127	0.622	0.241	1.47	–
Ref-based	354	0.654	0.198	1.49	–
Ref-free	596	0.670	0.145	1.48	12
SVA	123	0.644	0.091	1.23	6
ISVA	72	0.482	0.040	1.12	11
EWASher	1	0.052	0.030	0.92	–
CellCDec	169	0.598	0.008	1.27	–
Deconf	184	0.644	0.204	1.41	–
RUV	2943	0.654	0.228	1.93	33

*KS* Kolmogorov–Smirnov statistic, *GIF* genomic inflation factor

In the high-precision scenario, the NFP is extremely high when no adjustment is used. Most methods, however, perform quite well in reducing the GIF and KS statistics, reducing the NFP and retaining decent power (with the exceptions of Ref-free and EWASher as seen previously). In contrast, for the low-precision scenario, where there is much more variability from one individual to the next in the mixture proportions, as well as substantial differences between cases and controls, performance is generally poor. The QQ plots display substantial inflation, and most methods have very high NFP. Even the Ref-based method has very high NFP, and notably the QQ plot for RUV has enormous inflation and with the NFP at 2943. In fact, the unadjusted analysis appears to be one of the better choices here, with lower NFP and good power; ISVA also seems to perform better than the others.

### Scenario 6: continuous phenotype simulation results

In our simulation with continuous phenotypes, the relative performances of the methods are different again. Table 8 and Additional file 1: Figure S6 indicate that unlike all the other scenarios, the Ref-free method performs fairly decently in this case, leading to small reductions in the GIF and KS statistics and a small improvement in power. RUV’s performance is one of the best here, with a low NFP, good power, and an excellent GIF value. In contrast, the CellCDec method, which had performed quite well in all the other scenarios, shows extensive inflation across the QQ plots (GIF = 1.44) and a very high NFP. We were unable to obtain results for the Deconf method with three components within the available computational time limits on the Mammouth Compute Canada cluster. We note that the EWASher method does not allow continuous phenotypes and cannot be used in this scenario.

**Table 8:** Performance metrics under simulation scenario 6 (continuous)

Method	Number of false positives	Power	KS	GIF	$\hat {K}$
Unadjusted	135	0.532	0.081	1.37	–
Ref-based	13	0.498	0.031	0.99	–
Ref-free	125	0.618	0.061	1.17	14
SVA	29	0.502	0.006	0.99	10
ISVA	114	0.520	0.076	1.21	14
CellCDec	422	0.420	0.079	1.44	–
Deconf ^a^	–	–	–	–	–
RUV	16	0.550	0.006	0.966	39

*KS* Kolmogorov–Smirnov statistic, *GIF* genomic inflation factor

^a^No results were obtained with *K*=3 in the allowable time on the computational cluster Mammouth

### Scenario 7: simulation with a small number of true DMSs

**Fig. 3:**
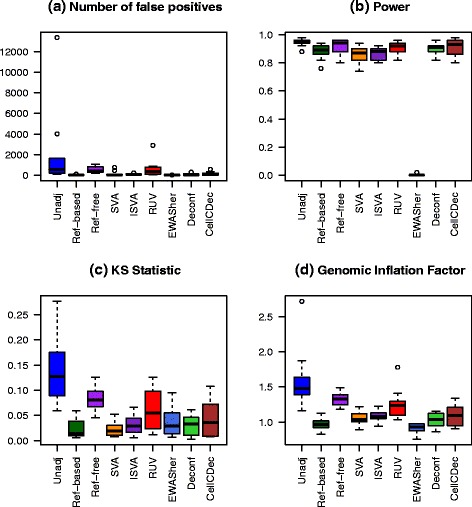
Comparison of performance metrics over ten replications in Scenario 7, few associated DMS. **a** The number of non-differentially methylated sites with a raw *p* value less than 10^−4^. **b** Power at significance level 10^−4^. **c** Kolmogorov–Smirnov statistic. **d** Genomic inflation factor

To consider cell-type mixture effects when there are only a few epistable alleles, as has been observed in several studies [29, 30], we created a simulation with only 50 DMSs, exhibiting moderate to strong positive associations with the phenotype in both cell types. Ten replications of the simulation were performed. The parameters in Table 1 were held constant over the different replications, except for *σ*_*jk*_, which was generated from a uniform distribution to induce some additional variation. Box plots comparing performance over the ten replications can be seen in Fig. 3. QQ plots for all methods under one of the replications can be seen in Additional file 1: Figure S7.

Unsurprisingly, the performance metrics are quite variable when the analysis does not adjust for cell-type composition. The NFP of both the reference-free method and RUV vary substantially. In contrast, both the reference-based and SVA methods show a very good reduction in the number of non-DMSs below this *p* value threshold across all replications. Most methods do not achieve a power as high as the unadjusted method; however, the lowered NFP is a worthy tradeoff for the loss in power, especially for the reference-based and SVA methods. Most methods improve the KS statistic and GIF, except for the reference-free method, which has values almost as high as those in the unadjusted analysis.

### Scenario 8: widespread, subtle, correlated DMS effects

**Fig. 4:**
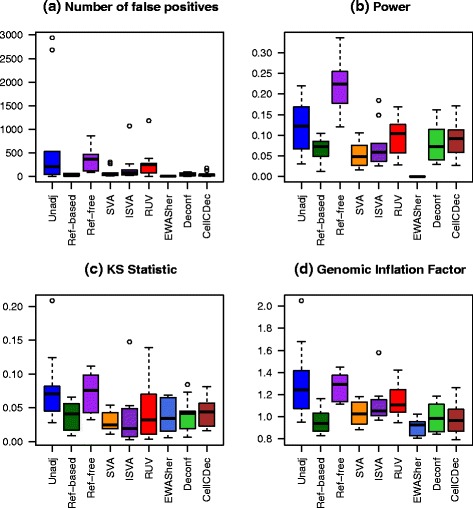
Comparison of performance metrics over ten replications in the many associated DMSs scenario. **a** Number of non-differentially methylated sites with a raw *p* value less than 10^−4^. **b** Power at significance level 10^−4^. **c** Kolmogorov–Smirnov statistic. **d** Genomic inflation factor

In the final simulation scenario, we simulated a large number of associated DMSs to capture the possibility of a subtle genome-wide shift in methylation, such as might be seen for a large change in metabolic functioning or in immune system function. Here, we randomly selected 10,000 CpG sites to be DMSs, and unlike the previous simulation scenarios, we created dependence between the phenotype-associated methylation shifts at these sites. The DMSs were grouped into two blocks of size 5000, and a moderate background level of correlation between the mean effect sizes *μ*_*jk*_ was included in each block. Effect sizes across blocks were still generated independently. Additional details of the dependence structure can be seen in step 4 of the “Methods” section, and the parameter choices can be seen in Table 2. Box plots comparing performance over the ten replications are shown in Fig. 4. QQ plots for all methods under one of the replications can be seen in Additional file 1: Figure S8.

In this scenario, NFP is less variable among the different adjustment methods, with the exception of the reference-free method. Once again, the reference-based and SVA methods do quite well, but Deconf and CellCDec also perform well in this scenario. Power is lower in all methods compared to the other simulation scenarios, with the exception of the reference-free method, though the high NFP for that method undermines this result. Most methods perform fairly similarly when considering the KS statistic and GIF.

### Estimated latent dimension

Our simulation is based on complex mixtures of methylation profiles from two separated cell types. It is, therefore, interesting to note that for all the methods that provide estimates for the latent dimension (the last column of Tables 3, 4, 5, 6, 7 and 8), these estimates are consistently much larger than 2. Estimates are obtained for the Ref-free, SVA, ISVA, and RUV methods. Both SVA and ISVA assume the number of surrogate variables is less than or equal to the number of true confounders whose linear space they span, and for RUV, the authors themselves commented that the estimated values for *K* do not necessarily reflect the true dimension [27]. All estimates are generally greater than ten, and RUV’s estimates tend to be over 30. In fact, there may be some additional sources of variation present in the original cell-separated methylation data, and these factors are likely being captured by these numerous latent variables. In fact, analyses of the original cell-type-separated data using patient age as the predictor resulted in estimated latent dimensions that were themselves large. For example, random matrix theory [31] (which is used for dimension estimation in the reference-free method and ISVA) estimated a latent dimension of ten for both T cells and monocytes when analyzed separately. Furthermore, SVA estimated the number of surrogate variables to be seven and nine for T cell and monocytes, respectively.

### Results from analysis of the ARCTIC data set

We tested the performance of these eight adjustment methods on 450K measurements from the Assessment of Risk in Colorectal Tumors in Canada (ARCTIC) study [32], and the methylation data are deposited in dbGAP under accession number [phs000779.v1.p1]. We analyzed only 977 control subjects from this study, restricting to those where DNA methylation was measured on lymphocyte pellets, and examined the association between smoking (ever smoked) and methylation levels at all autosomal probes who passed quality control (473,864 probes). We excluded the colorectal cancer patients from this analysis due to concerns that their methylation profiles may have been affected by treatment. Patient age was included as a covariate in all analyses.

**Fig. 5:**
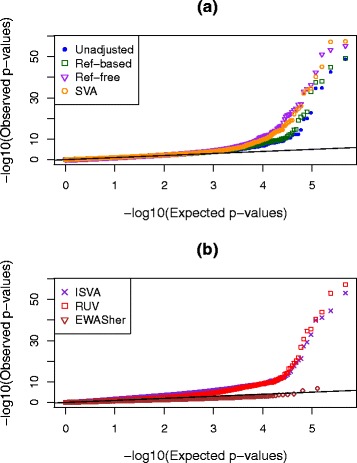
QQ plots of −log_10_ *p* values from the ARCTIC study with different adjustment methods. These are split across two panels, (**a**) and (**b**), to clarify the display

Figure 5 shows the QQ plots for seven adjustment methods, and Table 9 provides KS and GIF numeric metrics of performance. The Deconf and CellCDec methods could not be used with these data since the computational time exceeded the 5-day limit allowed on the Mammouth cluster of Calcul Quebec. As was seen in our simulations, the EWASher method seems to over-correct, leaving no significant probes, and the GIF is much smaller than 1.0. However, all other methods lead to QQ plots where the slope is larger after correction than before; the GIF estimates are substantially larger than for the unadjusted analysis. For SVA and ISVA, the KS statistic is increased after corrections are applied. Furthermore, among the top 1000 probes selected by each method (based on raw *p* value), none were shared by all methods (including unadjusted results). If EWASher was excluded, 87 probes overlapped among the most significant 1000, and 89 probes overlapped among methods excluding EWASher and the unadjusted results. Therefore, the methods are highlighting quite different results for the most significant probes.

**Table 9:** Performance metrics for the ARCTIC data with the most significant probes removed (top 5 %)

Method	KS statistic	GIF	$\hat {K}$
Unadjusted	0.0758	0.9142	–
Ref-based	0.0508	0.9907	–
Ref-free	0.0296	1.1499	32
SVA	0.0362	1.0962	15
ISVA	0.1208	1.6820	39
EWASher	0.7291	1.0164	–
RUV with three components	0.0906	1.2423	–

It was not possible to obtain results for the CellCDec and Deconf methods

*KS* Kolmogorov–Smirnov statistic, *GIF* genomic inflation factor

In Table 10, *p* values are shown for probes that have been linked to smoking status in a large published EWAS [33]. They specifically discuss seven CpGs, previously reported as associated with smoking, that were replicated in their work. Although substantial evidence of association can be seen at all probes, it is interesting to see the differences in significance across methods. For example, at probe cg21161138, significance ranges from 10^−7^ to 10^−25^.

**Table 10:** *P* values for sites previously found to be associated with smoking [33]

Site	Unadj	Ref-Based	Ref-free	SVA	ISVA	EWASher ^a^	RUV
cg06644428	2.83E-20	2.04E-21	1.96E-27	9.19E-27	4.41E-20	–	1.64E-22
cg05951221	2.72E-43	1.21E-45	5.41E-56	3.96E-58	3.20E-45	5.77E-01	1.05E-53
cg21566642	1.72E-49	6.93E-50	4.20E-54	6.59E-58	8.09E-54	6.45E-01	5.94E-58
cg01940273	2.09E-35	3.30E-38	4.07E-43	7.07E-46	6.93E-42	3.07E-01	1.45E-44
cg19859270	4.91E-13	2.52E-22	4.76E-35	2.21E-35	5.15E-28	–	2.16E-35
cg05575921	1.58E-35	7.79E-39	7.89E-52	5.65E-41	1.34E-40	–	3.43E-41
cg21161138	2.43E-07	2.99E-10	1.24E-25	1.68E-25	2.84E-21	9.05E-01	6.07E-21
cg06126421	1.78E-23	1.06E-33	7.38E-34	7.37E-33	9.69E-34	7.05E-01	2.55E-36
cg03636183	9.27E-21	6.74E-24	6.10E-37	1.51E-34	4.77E-30	3.09E-01	1.60E-31

^a^Several sites were filtered out by EWASher

### Results from analysis of the rheumatoid arthritis data set

We also performed analysis of data from a rheumatoid arthritis study published in 2013 [34]. The data are available from GEO (http://www.ncbi.nlm.nih.gov/geo/). Methylation was measured with the Illumina 450K array in whole-blood samples from 354 anti-citrullinated protein antibody-associated rheumatoid arthritis cases and 337 controls. The manuscript reported 51,478 CpGs as demonstrating evidence of significant association with disease status. Akin to the ARCTIC analysis, we compared the results of running the different cell-type adjustment methods on these data.

We attempted to replicate the original analysis as closely as possible by using the Illumina control probe scaling procedure, including the same covariates in our linear model (age, sex, and smoking status), and adjusting for cell-type composition using the reference-based method. However, when extracting the significant CpGs mentioned in their Additional file 1, the distribution of raw *p* values in our analysis did not match those in the original paper. This could suggest there is a step in the original analysis not explicitly stated in the paper’s “Methods” section. However, since for our purposes, we wish to compare the results of the adjustment methods relative to each other, we do not believe this to be a significant issue. For our comparison, we have used functional normalization [35]. Comparisons of the distribution of the reported *p* values and the ones we obtained can be seen in Additional file 1: Figure S9.

**Fig. 6:**
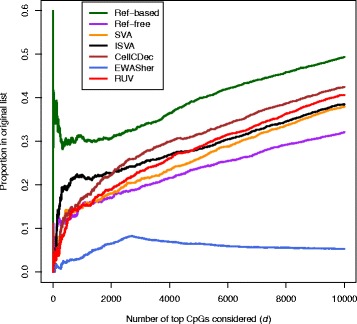
Agreement of the proportion of top *d* CpGs declared significant in the different cell-type adjustment methods with the top *d* CpGs in the originally reported list of significant CpGs from the rheumatoid arthritis study

The results from our analysis are summarized in Fig. 6. We performed the cell-type adjustment methods using all probes on autosomes, but restricted the analysis to the 51,478 probes reported in the paper. We examine the proportion of CpGs present in the top *d* significant CpGs found in each method that were also present in the top *d* CpGs found in the original paper. The method showing the highest concordance with the originally reported CpG list is the reference-based method, which was expected given that the reference-based method was used in the original analysis. It is evident, however, that the different adjustment methods do not replicate the findings of the original study, especially for smaller values of *d*. We have shown that the choice of cell-type adjustment method can drastically change the conclusions of an EWAS.

### Computational performance

To compare computational time across the different adjustment methods, we selected a random sample of 10,000 CpGs from the ARCTIC methylation matrix to create a benchmark data set. As we are not making any statistical inference here, all samples were included, regardless of whether we had matching cell-type sets or quality control status. Some of the methods calculate *p* values and parameter estimates internally, and others require the use of an external function to perform a linear fit. Therefore, to make the computational times comparable, we define the start time as when the adjustment method is first called, and the end time when all estimates and *p* values have been obtained.

**Fig. 7:**
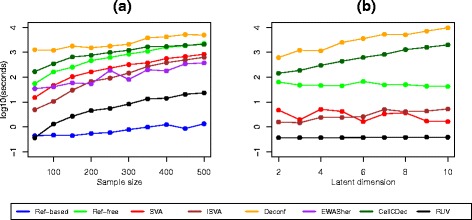
Computational time comparison. **a** Sample size. The latent dimension was estimated by the algorithms as needed. **b** Latent dimension. The sample size is fixed at 50

Figure 7 shows the running times on the log scale, as the sample size increases (*N*=50 to *N*=500), and for methods where a value of the latent dimension *K* can be specified, running times as *K* increases with a fixed sample size (*N*=50). There are major differences in running times for the cell-type adjustment methods. Not surprisingly, the Ref-based method is very fast, as is RUV. The slowest methods are Deconf and CellCDec. The computational time required for the Ref-free method also increases quickly with the sample size. In Fig. 7b, it is interesting to note that increasing *K* has very little effect on the speed of four of the six methods that require a specification of *K*. However, the computational times for both CellCDec and Deconf increase exponentially with larger values of *K*. As noted previously, we were not able to obtain results for these methods with the ARCTIC data set when using all autosomal probes. We note also that the complexity of preparation of the input files also varies from one algorithm to another.

## Discussion

We have presented an extensive comparison of eight different methods for adjusting for cell-type-mixture confounding, by designing a rich simulation based on cell-type-separated methylation data in SARD patients. Our simulation contained multiple levels of variability, between cell types, at the level of the probe means, and at the level of the individual. We found that there was no adjustment method whose performance was uniformly the best, and in fact in some of our scenarios, the unadjusted results were quite comparable to the best adjusted results. These general conclusions were similar whether we had a few DMSs of large effect (scenario 7) or many correlated DMSs of smaller effect size (scenario 8).

Ten replications were run in both simulation scenarios 7 and 8. However, for scenarios 1 through 6, the reported results were obtained from one run of the simulation for each scenario. There are two reasons for this: firstly, the computational time for some methods was very long, and hence, multiple simulations would be extremely time-consuming. More fundamentally, however, since our metrics of performance are calculated from the distributions of behavior across all good quality probes in the 450K array, we obtained very similar results from repeated runs of our simulations, during the phase when we were designing the simulation setup and choosing parameter values.

In many of our simulated scenarios, as might be expected, the reference-based method performed well. This method is very easy to implement, and as seen in the computing performance section, it runs very quickly, even on larger sample sizes. It usually achieved good statistical power, and, with one exception, reduced NFP relative to the unadjusted model. It also has the advantage of being able to estimate directly the cell-type composition of each sample. Therefore, the Ref-based method is an obvious choice when a complete set of the required cell-separated methylation profiles are available; however, this is not always the case. For some tissues, cell types that are of particular interest are very difficult or impossible to extract; one example here would be the syncytiotrophoblast cells in placenta [36, 37].

In every case we examined, EWASher did a very good job in reducing *p* value inflation, and GIF values were substantially reduced from the unadjusted analyses. However, that this method so strictly forces the GIF factor downwards may raise concerns about over-correction. If there were, for example, global hypermethylation associated with a disease, adjustment using EWASher would be overly conservative. Additionally, part of the algorithm involves filtering out loci that are unilaterally high or low among all subjects. The assumption behind this filter is that these loci are, for all intents and purposes, completely methylated or unmethylated and any associations between these probes and the phenotype are not interesting. This may be an overly strong assumption. In our simulations, this filter results in the dramatically worse power of this method, since we did not restrict the randomly selected DMS loci to any particular mean level of methylation. Furthermore, the EWASher method is quite difficult to implement. Although most methods can be run in R (https://www.r-project.org), EWASher requires the user to create three separate input files for a standalone executable, and then to perform post-processing in R.

We cannot explain the poor performance in our simulations of the Ref-free method. The NFP were almost always more inflated than in the raw data, and this inflation is clearly visible in the QQ plots. Furthermore, the implementation was somewhat more complex since the approach involved one step to estimate the latent dimension, a second to get parameter estimates, and then finally bootstrap calculations to obtain standard errors. The performance of the Ref-free method was good for scenario 6 with a continuous phenotype, so we hypothesize that there are some linearity assumptions in the correction that are being violated in our binary phenotype simulations.

The performance of CellCDec and Deconf was generally quite good for binary phenotypes. The CellCDec method exists as a C++ program, and was quite easy to implement. The number of latent cell types must be specified in advance, which is a limitation. The run time was longer for this algorithm than the others, and increased quickly with the assumed number of cell types; in fact, we were unable to obtain results with the ARCTIC data. CellCDec does not use phenotype information; it would be interesting to see how this program performs if it took the phenotype and other covariates into account. For Deconf, the most important limitation was the running time. In all cases, it took longer to run than the other adjustment methods, and we were unable to obtain results for the ARCTIC data. The run time was sensitive to both increases in sample size and number of cell types. Akin to CellCDec, that it does not internally estimate the number of cell types is an issue.

The results for ISVA and RUV were often among the better ones with a couple of notable exceptions: NFP were extremely high for RUV in the low-precision scenario, and for ISVA in the no confounding scenario. The computational time for the ISVA method also increased quite rapidly with sample size. RUV is very easy to run and is available as an R function. It contains a function to estimate the latent dimension (*K*), although, akin to the other methods that estimate *K*, the estimated dimension tends to be much higher than the simulated reality. We performed some investigations into how RUV performs at a range of values for *K*, and the best performance was observed, in most simulation scenarios, at smaller values such as *K*=3. Recently, Houseman also found that estimated latent dimensions obtained through random matrix theory may not be the best choices [38]. RUV is also extremely fast, slower only than the Ref-based method, and as shown in Fig. 7, the computational time is essentially invariant as the latent dimension is varied, making this an attractive option. Nevertheless, the SVA method, although rarely the best, did not have any notable failures across our scenarios, and was easy to implement.

There are other methods for deconvolution that we did not examine, especially in the computer science and engineering literature [39]. However, it is not clear whether these methods would be easily adapted for use on methylation data. Also, new methods for DNA methylation analysis continue to be published, such as [40]. However, the spectrum of methods that we have examined includes the most-commonly used approaches. All methods that we have examined assume approximately linear relationships between the phenotype and the methylation levels or covariates; however, this should not be an important limitation since approximate linearity should hold [38].

The latent dimension, when estimated, was rarely similar to the dimension of *K*=2 implemented in our simulation. However, these estimates of *K* capture aspects of heterogeneity in the data that are only partially attributable to the mixture of data from two cell types. This heterogeneity may also be partially due to technological artefacts from batch effects or experimental conditions, and in particular to the fact that subtler cell lineage differences will still be present even after cell sorting [38].

In summary, our simulation study comparing methods found a wide range of performance across our scenarios with notable failures of some methods in some situations. We recommend SVA as a safe approach for adjustment for a cell-type mixture since it performed adequately in all simulations with reasonable computation time. In all situations, EWAS results are extremely sensitive to the normalization and cell-type adjustment methods used, and hence, this issue should receive more attention when interpreting findings.

A set of scripts enabling implementation of all these methods can be found at https://github.com/GreenwoodLab/CellTypeAdjustment.

## Conclusions

We have compared eight different methods for adjusting methylation data for cell-type-mixture confounding in a rich and multi-layered simulation study, and in a large set of samples where methylation was measured in whole blood. No method performs best in all simulated scenarios, nevertheless we recommend SVA as a method that performed adequately without notable failures.

## Methods

### Patient data and quality checks

Ethical approval was obtained at the Jewish General Hospital and at McGill University, Montreal, QC, to obtain whole-blood samples from the patients with SARDs, at the time of initial diagnosis prior to any treatment. Cell purification and phenotyping protocols for cell subset isolation, analysis, purity evaluation, fractionation, and storage were standardized and optimized. Then 40 ml of peripheral blood were obtained from the above subjects and processed within 4 hours. PBMCs were separated with lymphocyte separation medium (Mediatech, Inc.). Isolated PBMCs were sequentially incubated with anti-CD19, anti-CD14, and anti-CD4 microbeads (Miltenyi Biotec). Automated cell separation of specific cell subpopulations was performed with auto-MACS using positive selection programs. An aliquot of the specific isolated cell subtypes was used for purity assessment with flow cytometric analysis. A minimum of 2 million cells from each subpopulation with a purity higher than 95 % were frozen in liquid nitrogen for the epigenomic studies. The optimized protocols required the isolation of sufficient numbers of CD4 ^+^ lymphocytes (9.04±4.03×10^6^) and CD14 ^+^ monocytes (7.89±2.96×10^6^), and CD19 ^+^ B lymphocytes (2.02±1.42×10^6^), of sufficient purity to perform the epigenetic analyses.

The required number of cells with the right purity was not always available, especially for the CD19 ^+^ B lymphocytes, so we did not have all three cell types for all patients; for this reason, the simulation used only two cell types and 46 patients. Illumina Infinium HumanMethylation450 BeadChip data were normalized with funnorm [35]. Also, a number of probes were removed, specifically those on the sex chromosomes as well as probes close to single-nucleotide polymorphisms [41]. There were 375,639 probes remaining after filtering.

### Details of the simulation method

This simulation design was initially developed in the master’s thesis of the first author [42].

1. Selection of DMS probes: *S*=500 probes were randomly selected to be associated with the phenotype.
2. Phenotype (*z*_*i*_, *i*=1,…,*n*): A random sample of size 46 was drawn from either a Bernoulli distribution (*p*=0.5) for a binary phenotype, or from a standard normal distribution for a continuous phenotype.
3. Cell-type-specific methylation values for non-DMS probes: Let *β*_*ijk*_ represent the true methylation value for individual *i*, probe *j*, and cell type *k*. For a probe that is not a DMS probe, the simulated value $\beta ^{\prime }_{ijk} = \beta _{ijk}$.
4. Cell-type-specific methylation values for DMS probes:
  Cell-type-specific means are sampled from normal distributions with given parameters. That is, for chosen values *μ*_*k*_ and *σ*_*k*_ for *k*=1,2, cell-type-specific means that for each DMS probe, *μ*_*jk*_ are generated from $\mu _{jk} \sim N\left (\mu _{k}, {\sigma ^{2}_{k}}\right)$. In scenario 8, we split the DMSs into two blocks and simulate effect sizes from a multivariable normal distribution with a fixed background correlation in each block. That is, probes in the same block are correlated, but probes across blocks are independent from one another. In this scenario, the parameters *μ*_*k*_ for *k*=1,2 differ between the blocks.
  The simulated cell-type-specific methylation effect, *ε*_*ijk*_, at a DMS, for an individual sample *i* and an individual probe *j*, is another random quantity, so that
    $$ e_{ijk} \sim N\left(\mu_{jk}, \sigma^{2}_{jk}\right)   $$
    where *σ*_*jk*_ is a parameter provided to the simulation.
  For either a binary or continuous phenotype *z*_*i*_, the simulated methylation value *β*_*ijk*_ is then
    $$ \beta^{\prime}_{ijk} = \text{logit}^{-1} \left(\text{logit} \left(\beta_{ijk} \right) + z_{i} e_{ijk} \right).   $$
    Although all the random effects were simulated on a linear scale, the results are reconverted to the (0,1) scale since several of the cell-type adjustment methods require this range.
5. Combining across cell types:
  Each individual is assumed to have a unique mixture of the two cell types in a way that depends on the phenotype, *z*. Let $\alpha ^{(0)} = \left (\alpha _{1}^{(0)}, \alpha _{2}^{(0)}\right)^{\top }$ represent the average proportions of the two cell types when *z*_*i*_=0, and then let $\alpha ^{(Z)} = \left (\alpha _{1}^{(Z)}, \alpha _{2}^{(Z)}\right)^{\top }$ be these proportions when *z*_*i*_=*Z*. We then say,
    (1) $$ \begin{aligned} \alpha^{(Z)} &\!= \alpha^{(0)}+ Z \\ &\!\quad\times\!\! \left[\begin{array}{l} \text{\!average change in proportion in monocytes\!\!} \\ \text{\!average change in proportion in T cells}\! \end{array}\right]\!. \end{aligned}  $$
  The cell-type proportions *p*_*ik*_ for individual *i* were then generated from $\text {Dirichlet}\left (\rho \alpha _{k}^{(Z)}\right)$, where *ρ*>0 is a precision parameter, such that larger precision corresponds to less variation in the observed values.
  The final simulated beta value for person *i* at CpG site *j* becomes
    (2) $$ \beta^{f}_{ij} = p_{i1} \beta^{\prime}_{ij1} + p_{i2} \beta^{\prime}_{ij2}.  $$

Key notation definitions are summarized in Table 1, and parameter choices for the simulations are in Table 2.

### Description of adjustment methods

The performance of eight popular methods is compared. Brief descriptions of each method are provided here, and Table 11 compares some key features of the methods, including some details of the implementations. This set of eight methods is not an exhaustive list of all methods available at this time. In fact, in other fields, particularly engineering and computer science, there exists a plethora of other methods under the guise of deconvolution providing the same kind of correction for unmeasured confounding in other high-throughput data sources [39]. However, we include and compare many of the approaches that are in common usage in the last few years in the world of genomics and epigenomics.

**Table 11:** Comparison of some features of the methods for cell-type mixture adjustment

Method	Phen. allowed ^a^	Input values ^b^	*K* ^c^	Link
Ref-based	Any	Beta	N/A	http://people.oregonstate.edu/~housemae/software/TutorialLondon2014
				http://bioconductor.org/packages/release/bioc/html/minfi.html
Ref-free	Any	Beta	Estimated	http://cran.r-project.org/web/packages/RefFreeEWAS/index.html
SVA	Any	Beta or logit(beta)	Estimated	http://bioconductor.org/packages/release/bioc/html/sva.html
ISVA	Continuous	Beta or logit(beta)	Estimated	http://cran.r-project.org/web/packages/isva/index.html
EWASher	Binary	Beta	Estimated	http://research.microsoft.com/en-us/downloads/472fe637-7cb9-47d4-a0df-37118760ccd1
CellCDec	Not used	Beta	Input	https://github.com/jameswagner/CellCDec
Deconf	Not used	Beta or logit(beta)	Input	http://web.cbio.uct.ac.za/~renaud/CRAN
RUV	Any	Beta or logit(beta)	Estimated	https://cran.r-project.org/web/packages/ruv/index.html

^a^What kinds of phenotype are allowed?

^b^Does the method use methylation proportions (beta values)? Or logit transformed beta values?

^c^Does the method estimate the number of latent cell types *K*, or is *K* input into the algorithm?

#### Reference-based

This method was published in 2012 by Houseman et al. [19]. It relies on the existence of a separate data set containing methylation measurements on separated cell types. The method uses methylation profiles for the individual cell types to estimate directly the cell-type composition of each sample. However, cell-separated data are not always available for all constituent cell types.

#### Reference-free

The second method from Houseman et al. does not depend on a reference data set, and therefore, can be used in methylation studies on any tissue type [20]. Rather than directly estimating cell-type composition, the reference-free method performs a singular value decomposition on the concatenation of the estimated coefficient and residual matrices from an initial, unadjusted model. A set of latent vectors is then obtained that accounts for cell type in further analyses.

#### Surrogate variable analysis

Surrogate variable analysis (SVA) is a popular method that was introduced by Leek and Storey in 2007 [23]. It was not specifically intended for use in methylation studies, but is nonetheless well suited for such analyses. SVA seeks a set of surrogate variables that span the same linear space as the unmeasured confounders (i.e. cell-type proportions). It is based on a singular value decomposition on the residual matrix from a regression model not accounting for cell-type composition. The total number of surrogate variables included in the model is based on a permutation test.

#### Independent surrogate variable analysis

Independent surrogate variable analysis (ISVA) from Teschendorff et al. [24] is very similar in principle to SVA. The main difference is that instead of applying singular value decomposition, it uses independent component analysis (ICA), which attempts to find a set of latent variables that are as statistically independent as possible.

#### FaST-LMM-EWASher (EWASher)

This method from Zou et al. [22] extends the Factored Spectrally Transformed Linear Mixed Model algorithm (FaST-LMM) [43] for use in the context of EWAS. A similarity matrix is calculated based on the methylation profiles, and principal components are subsequently included in the linear mixed model until GIF is controlled. The maximum number of principal components allowed was fixed as ten.

#### Removing unwanted variation

The method called Removing Unwanted Variation (RUV) was published in 2012 [26] by Gagnon-Bartsch and Speed. It performs a factor analysis on negative control probes to separate out variation due to unmeasured confounders, while leaving the variation due to the factors of interest intact. Here we use RUV-4, an extension to the original published version, which uses elements from RUV as well as SVA [27]. Control probes were chosen from a list of 500 probes on the 450K platform known to be differentially methylated with blood cell type and age [13]. We selected probes that were not strongly correlated with the simulated phenotype.

#### Deconfounding

The Deconf method from Repsilber et al. [25] was developed for gene expression studies on heterogeneous tissue samples, but is applicable for use in EWAS. The algorithm performs a non-negative matrix factorization on the methylation matrix, but does not consider the phenotype in correcting for the heterogeneity and does not estimate the number of cell types present.

#### CellCDec

CellCDec was developed by Wagner [21], and is similar to Deconf in that it does not consider the phenotype in performing its decomposition and does not internally estimate the number of cell types present. The method assumes a specific regression parameterization, and makes random perturbations to the model parameters, which are accepted if there is a decrease in the sum squared residuals.

#### Additional statistical details

For each of the simulation scenarios 1–6, the simulation was run once, whereas in scenarios 7 and 8, we performed ten replications. DMSs were chosen randomly in each simulation scenario and within each replication. After cell-type adjustment, a linear model was performed including the latent variables as covariates (except in EWASher where the model was run within the function call). Standard errors were obtained after performing the empirical Bayes method eBayes from the limma package in R. Probes were declared significant if the *p* value fell below the fixed value 10^−4^.

In the reference-free method, standard errors were obtained via the bootstrap procedure included in the method. To estimate standard errors, 100 bootstrap samples were generated. We performed test runs with higher numbers of bootstrap samples (500 and 1000), but did not find significant differences in the resulting *p* values.

For SVA, we used the “iteratively re-weighted least squares” option; however, no control probes were specified. For RUV, the control probes were chosen from sites previously shown to be associated with blood cell type and age, but were not significantly associated with the phenotype of interest, a necessary condition for a probe to be used as a control in this method.

For each of the methods requiring a pre-specified value of *K*, the latent dimension, we ran the methods multiple times with different values of *K*. We consistently found that performance did not change with increasing values of *K* and so when running the methods CellCDec and Deconf, the value of *K* was fixed at 3 in all scenarios to keep running times down. For the RUV method, we also noticed better performance when *K* was fixed at 3, despite that the method consistently estimated a much higher value for that parameter. All results shown for RUV were run with *K* fixed at 3.

None of the other adjustment methods had specific options or tuning parameters that needed to be provided by the user.

## Compliance with ethical standards

Ethics committee approval for this study was obtained at McGill University and all subjects provided informed written consent to participate in the study. The Institutional Research Board (IRB) number is [A12 M83 12A]. This study complies with the Helsinki Declaration.

## Availability of supporting data

The data sets supporting the results of this article are available in the repositories:

1. ARCTIC data are in dbGAP under accession number [phs000779.v1.p1], http://www.ncbi.nlm.nih.gov/projects/gap/cgi-bin/study.cgi?study_id=phs000779.v1.p1.
2. The rheumatoid arthritis data are available under accession number [GSE42861], https://www.ncbi.nlm.nih.gov/geo/query/acc.cgi?acc=GSE42861.
3. Data for simulation scenarios for cell-type mixtures are available at Zenodo [10.5281/zenodo.46746], https://zenodo.org/record/46746%23.VtW8H2SAOko.

## Additional file

Additional file 1 Contains plots showing simulated effect sizes among 500 CpGs in simulation scenario 1, and QQ plots for *p* values of several simulation scenarios. (PDF 771 kb)
