# Supplementary material for: An evaluation of methods correcting for cell-type heterogeneity in DNA methylation studies
Source: Genome Biol. 2016 May 3;17:84. doi: 10.1186/s13059-016-0935-y (PMC4855979; doi:10.1186/s13059-016-0935-y)

Supplementary figures:

Figure S1. Histograms of simulated effect means μ_{jk} for truly associated probes in Simulation Scenario 1

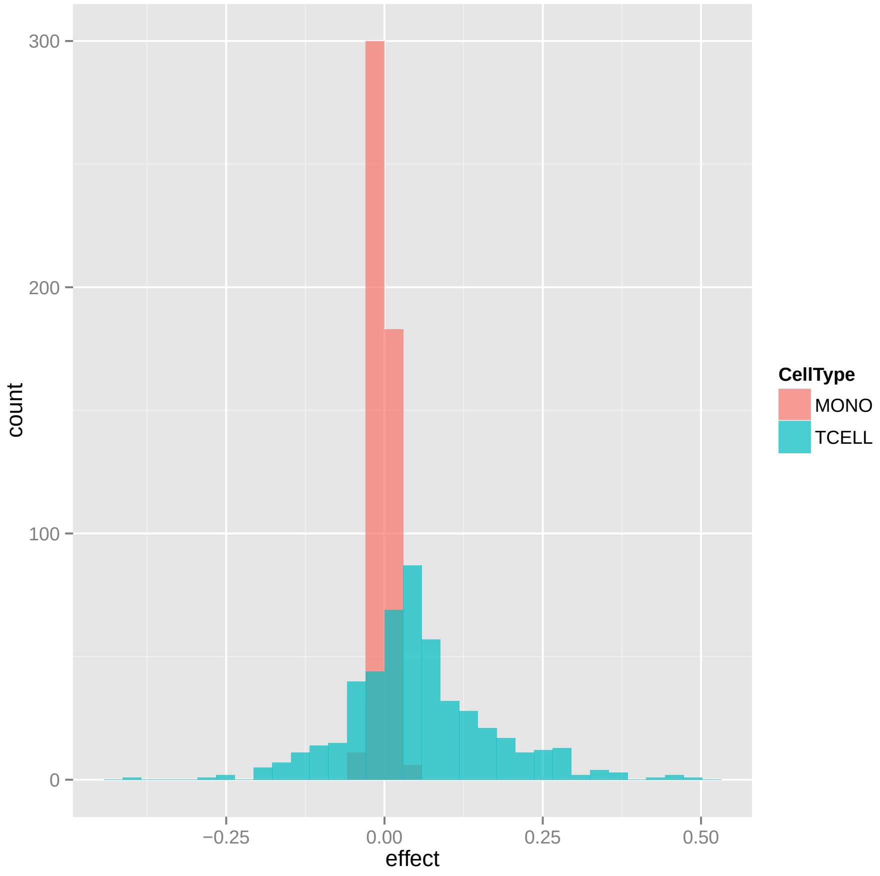

Figure S2: QQ-plots showing distributions of p-values in when there is no confounding (Simulation Scenario 2).

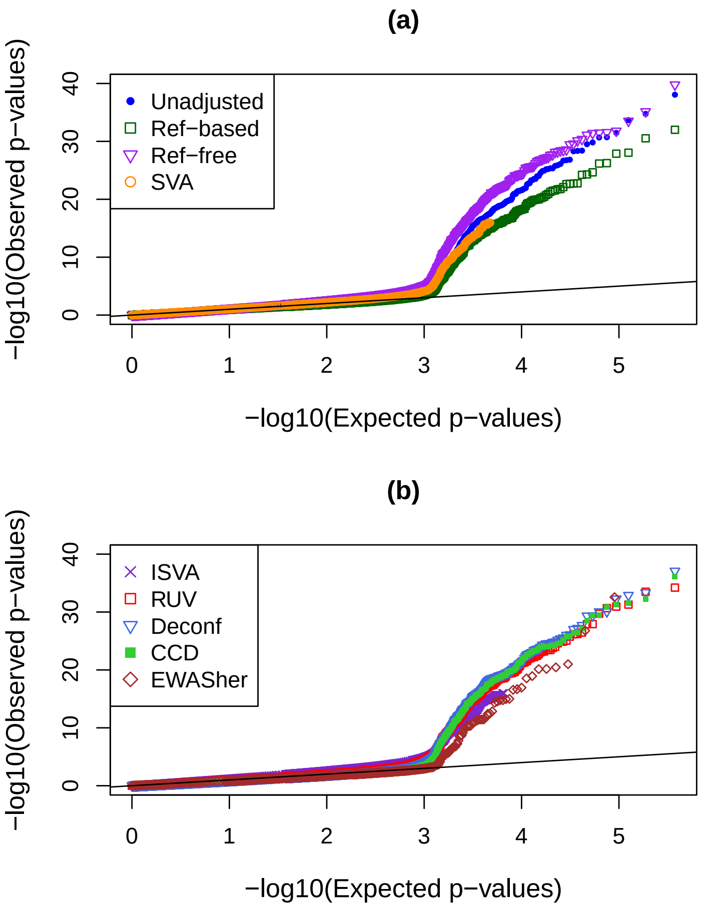

Figure S3: QQ-plots showing distributions of p-values in when there are opposite effects (Simulation Scenario 3).

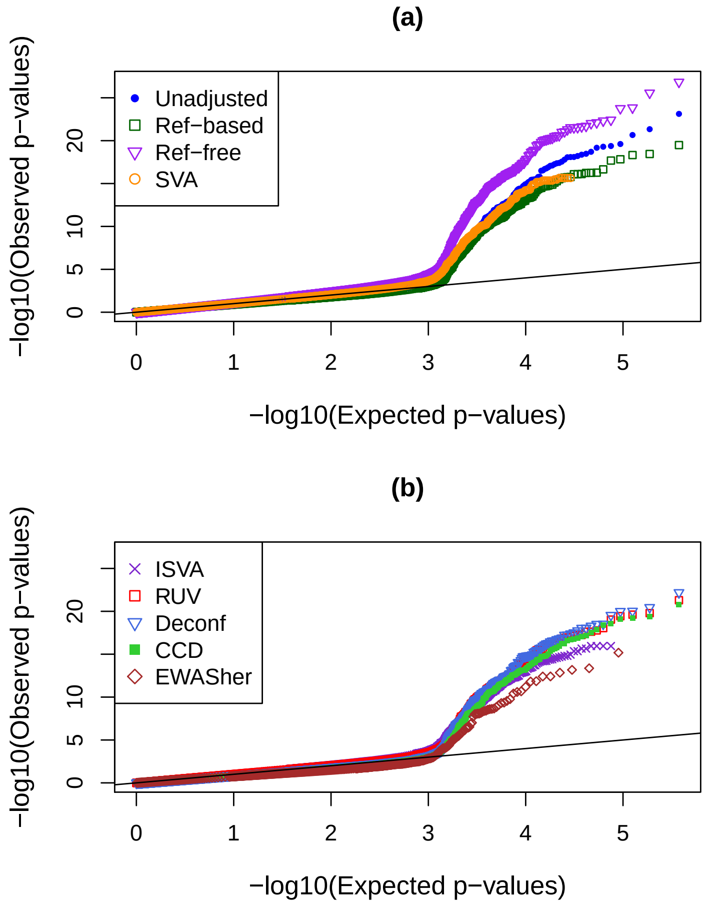

Figure S4: QQ-plots showing distributions of p-values in when there is high precision (Simulation Scenario 4).

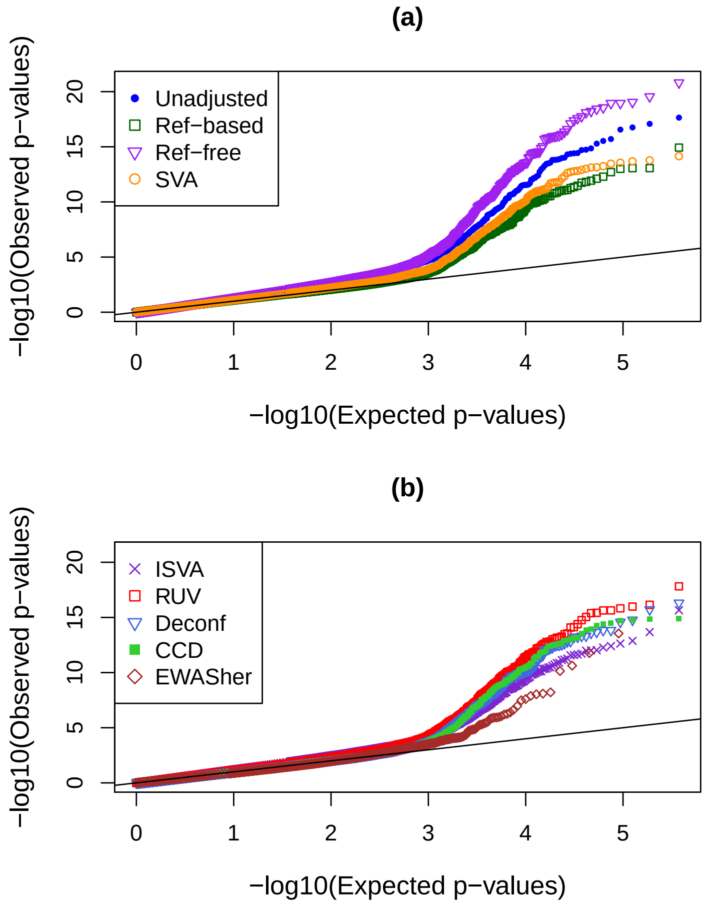

Figure S5: QQ-plots showing distributions of p-values in when there is low precision (Simulation Scenario 5).

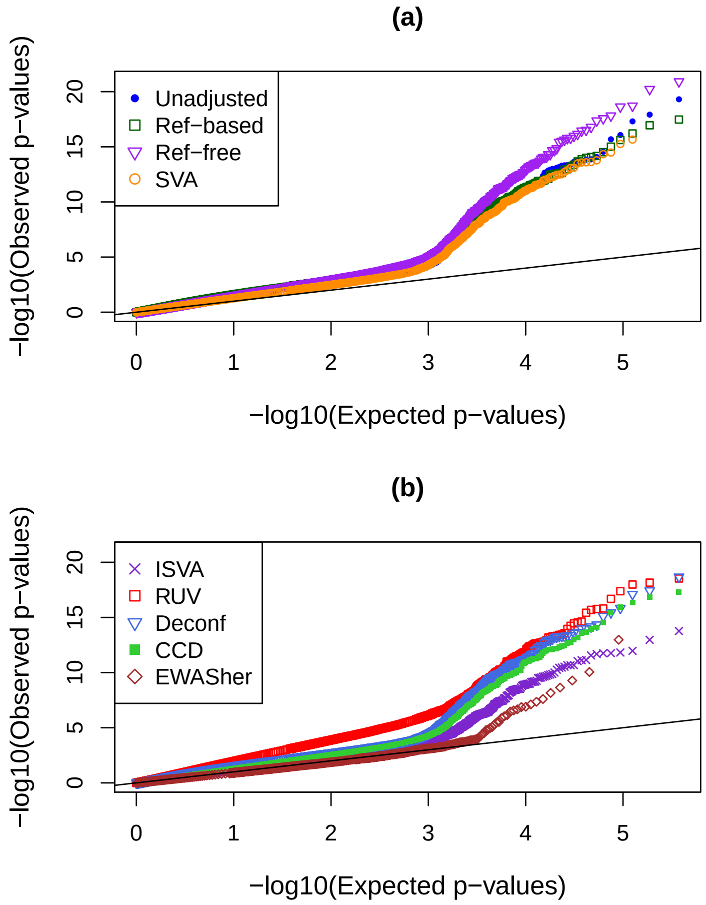

Figure S6: QQ-plots showing distributions of p-values for continuous phenotypes (Simulation Scenario 6).

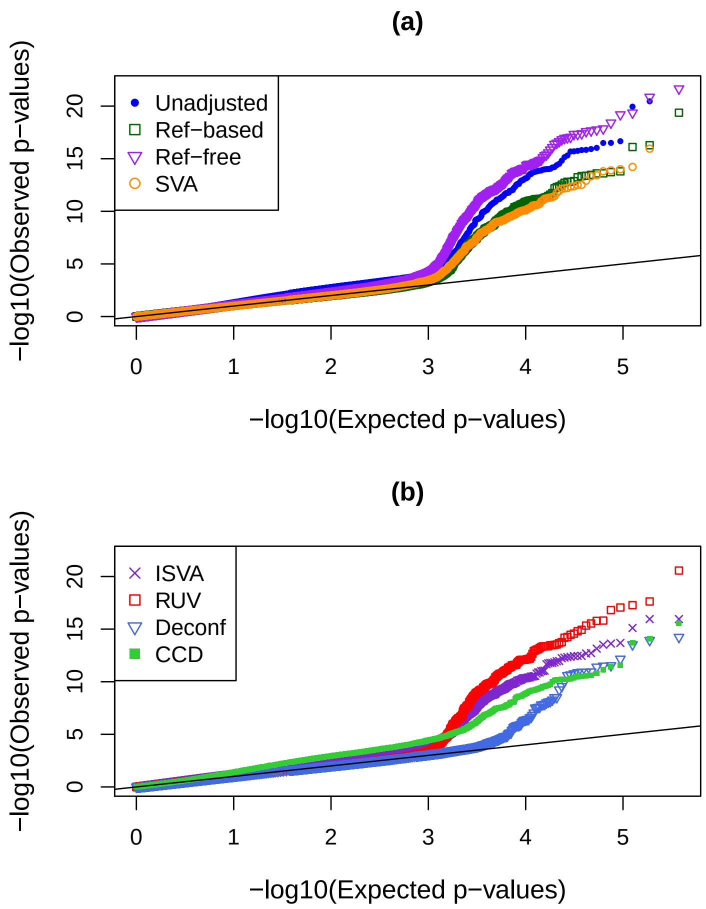

Figure S7: QQ-plots showing distributions of p-values for one replication of the few associated DMS scenario (Simulation Scenario 7).

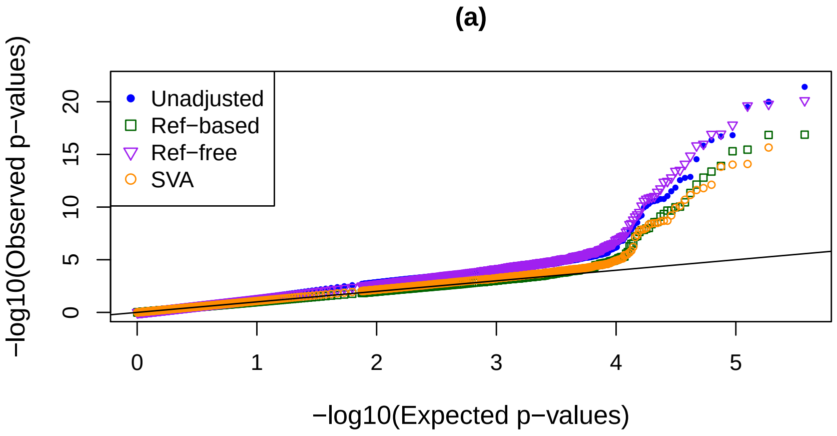

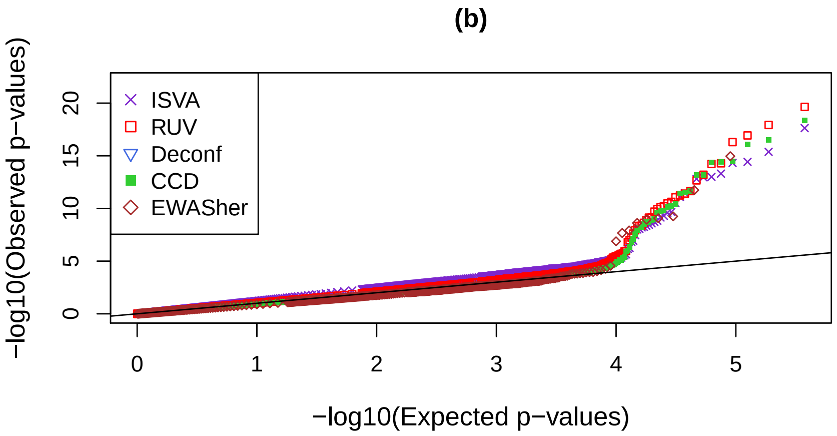

Figure S8: QQ-plots showing distributions of p-values for one replication of the many associated DMS scenario (Simulation Scenario 8).

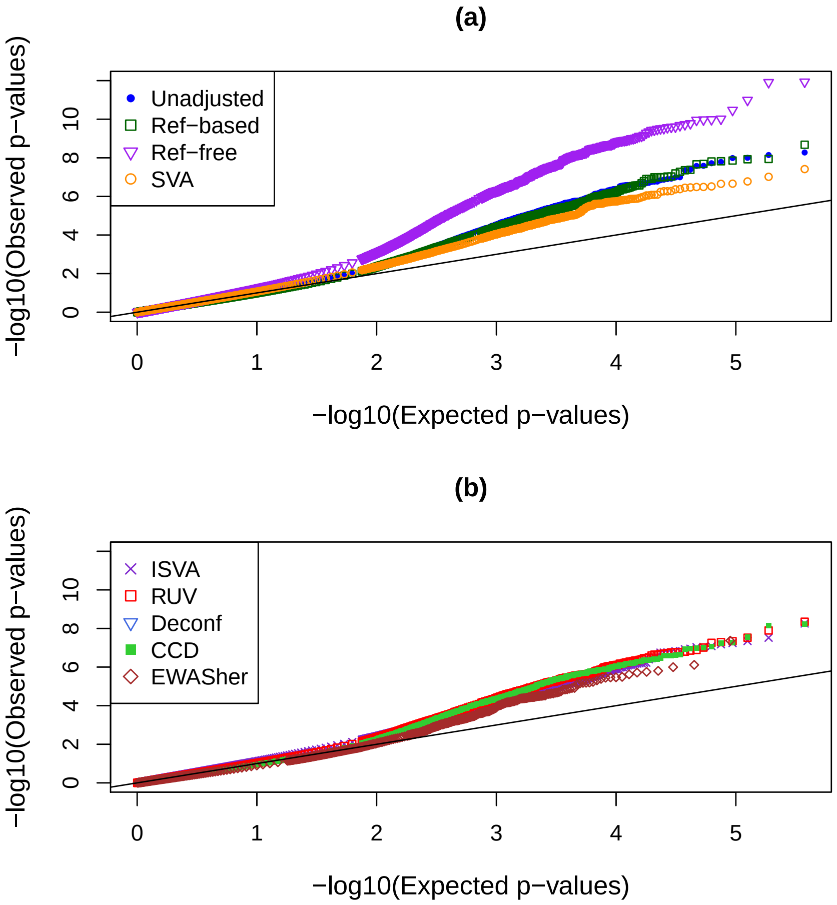

Figure S9: Distribution of p-values reported in rheumatoid arthritis EWAS is shown in red. The distribution found in our analysis comprising the same CpG sites is shown in blue. The different histograms represent different normalization methods in our analysis: (a) Illumina control probe scaling, (b) Illumina without control probe scaling, (c) Functional normalization, (d) Raw data (no normalization)

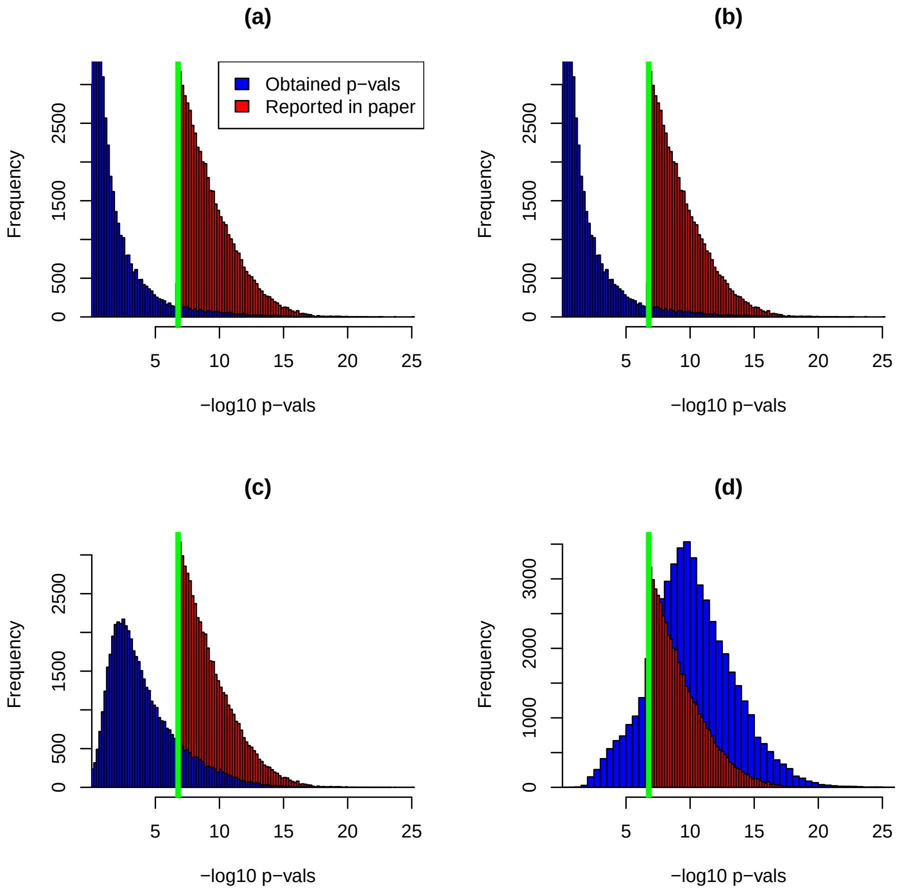

Supplement: Additional file 1 — Contains plots showing simulated effect sizes among 500 CpGs in simulation scenario 1, and QQ plots for p values of several simulation scenarios. (PDF 771 kb) [file 13059_2016_935_MOESM1_ESM.pdf]
